# PGC‐1*α* and fasting‐induced PDH regulation in mouse skeletal muscle

**DOI:** 10.14814/phy2.13222

**Published:** 2017-04-10

**Authors:** Anders Gudiksen, Henriette Pilegaard

**Affiliations:** ^1^ Section for Cell Biology and PhysiologyAugust Krogh BuildingDepartment of BiologyUniversity of CopenhagenCopenhagenDenmark

**Keywords:** Fasting, metabolism, PDH, PGC‐1*α*, skeletal muscle, substrate utilization

## Abstract

The purpose of the present study was to examine whether lack of skeletal muscle peroxisome proliferator‐activated receptor gamma coactivator 1 alpha (PGC‐1*α*) affects the switch in substrate utilization from a fed to fasted state and the fasting‐induced pyruvate dehydrogenase (PDH) regulation in skeletal muscle. Skeletal muscle‐specific PGC‐1*α* knockout (MKO) mice and floxed littermate controls were fed or fasted for 24 h. Fasting reduced PDHa activity, increased phosphorylation of all four known sites on PDH‐E1*α* and increased pyruvate dehydrogenase kinase (PDK4) and sirtuin 3 (SIRT3) protein levels, but did not alter total acetylation of PDH‐E1*α*. Lack of muscle PGC‐1*α* did not affect the switch from glucose to fat oxidation in the transition from the fed to fasted state, but was associated with lower and higher respiratory exchange ratio (RER) in the fed and fasted state, respectively. PGC‐1*α* MKO mice had lower skeletal muscle PDH‐E1*α*, PDK1, 2, 4, and pyruvate dehydrogenase phosphatase (PDP1) protein content than controls, but this did not prevent the fasting‐induced increase in PDH‐E1*α* phosphorylation in PGC‐1*α* MKO mice. However, lack of skeletal muscle PGC‐1*α* reduced SIRT3 protein content, increased total lysine PDH‐E1*α* acetylation in the fed state, and prevented a fasting‐induced increase in SIRT3 protein. In conclusion, skeletal muscle PGC‐1*α* is required for fasting‐induced upregulation of skeletal muscle SIRT3 and maintaining high fat oxidation in the fasted state, but is dispensable for preserving the capability to switch substrate during the transition from the fed to the fasted state and for fasting‐induced PDH regulation in skeletal muscle.

## Introduction

Skeletal muscle plays a major role in lipid and carbohydrate (CHO) utilization and is instrumental in maintaining metabolic flexibility with various metabolic challenges. Specifically fasting robustly prompts the protection of CHO stores through a coordinated decrease in skeletal muscle glucose oxidation and a switch toward fat oxidation. Pyruvate dehydrogenase (PDH) is thought to be a key element in such regulation of substrate utilization (Randle et al. 1994). Hence, the pyruvate dehydrogenase complex controls the access of CHO to the TCA cycle by irreversibly converting pyruvate to acetyl CoA thereby bridging glycolysis and oxidative substrate utilization (Harris et al. 2002). However, the detailed regulation of PDH during energy deprivation is not fully understood and merits further investigation.

The main regulation of PDH activity is thought to be through inhibitory phosphorylation by PDH kinases (PDKs) and activating dephosphorylation by PDH phosphatases (PDPs) at four known serine residues on the catalytic subunit PDH‐E1*α* (PDH) (Patel and Korotchkina 2001; Roche et al. 2001; Harris et al. 2002; Kiilerich et al. 2010). In addition, recent studies point toward other important posttranslational modifications as the mitochondrially located deacetylase Sirtuin 3 (SIRT3) has been reported to regulate the acetylation state of PDH‐E1*α* and thus the activity of PDH (Jing et al. 2013; Fan et al. 2014; Ozden et al. 2014). Previous studies have demonstrated that fasting reduces the activity of PDH in the active form (PDHa activity) (Sugden et al. 2000; Spriet et al. 2004), increases PDH^Ser293^ and PDH^Ser300^ phosphorylation (Kiilerich et al. 2010), and increases PDH‐E1*α* acetylation as well as PDK4 mRNA (Wu et al. 1999; Sugden et al. 2000; Pilegaard et al. 2003) and protein (Sugden et al. 2000; Spriet et al. 2004; Kiilerich et al. 2010) in rodent skeletal muscle. Furthermore, fasting has been reported to elicit a muscle‐type‐dependent regulation of PDK activity and PDK4 expression in rat skeletal muscle (Sugden et al. 2000). However, the factors determining this are not resolved.

Peroxisome proliferator‐activated receptor gamma coactivator 1‐alpha (PGC‐1*α*), first discovered and identified as a PPAR*γ*‐binding protein in brown adipose tissue (Puigserver et al. 1998), is a transcriptional coactivator that has been established to be an important regulator of mitochondrial biogenesis. Hence, muscle‐specific PGC‐1*α* overexpression mice have been shown to have increased content of oxidative proteins and conversely whole‐body PGC‐1*α* knockout and muscle‐specific PGC‐1*α* knockout mice to have lowered content of oxidative proteins in skeletal muscle (Lin et al. 2004; Leick et al. 2008; Geng et al. 2010). Furthermore, muscle‐specific PGC‐1*α* overexpression mice have been shown to exhibit lower respiratory exchange ratio (RER) during treadmill running (Calvo et al. 2008; Wong et al. 2015). This may be due to the enhanced skeletal muscle oxidative capacity of these mice (Lin et al. 2002), but may also be related to metabolic flexibility and regulation of substrate use. In accordance, the level of skeletal muscle PDH‐E1*α* protein content has been shown to follow differences in the level of muscle PGC‐1*α* in mice (Kiilerich et al. 2010) and PGC‐1*α* to regulate PDK4 expression in mouse skeletal muscle (Wende et al. 2005; Calvo et al. 2008; Kiilerich et al. 2010). In addition, the fasting‐induced downregulation of PDHa activity was blunted in PGC‐1*α* KO mice (Kiilerich et al. 2010) supporting that PGC‐1*α* plays a role in PDH‐mediated metabolic regulation during fasting. However, the impact of PGC‐1*α* on the switch from CHO to fat utilization during the transition from the fed to the fasted state, and on the level of fat utilization in the fasted state as well as the associated regulation of PDH in skeletal muscle is not fully resolved.

Therefore, the aim of the present study was to examine whether lack of muscle PGC‐1*α* (1) affects the time course of the switch in substrate utilization during the transition from a fed to fasted state, (2) affects regulation of fasting‐induced changes in PDHa activity, PDH phosphorylation, and PDH acetylation in skeletal muscle.

## Methods

### Animals

Generation of PGC‐1*α* MKO mice used in the present study was carried out as described previously (Lin et al. 2004; Geng et al. 2010). The mice were genotyped using PCR‐based muscle and tail genotyping as previously described (Leick et al. 2008) and the genotype was confirmed based on determination of PGC‐1*α* mRNA in muscle tissue after euthanization. Animals were kept on a 12:12‐h light–dark cycle at 22°C with ad libitum access to water and chow diet (Altromin 1314F, Brogaarden, Lynge, Denmark) until the intervention of the experiment. All experiments were approved by the Danish Animal Experiments Inspectorate and complied to the European Convention for the protection of vertebrate animals used for experiments and other scientific purposes (Council of Europe no. 123. Strasbourg, France 1985).

### Fasting procedure

At 3 months of age, female mice were individually housed for 3 days and subsequently allocated to either a fed group (FED), which maintained ad libitum access to both food and water, or a fasted group (FAST), for which the food was removed at 6 am and only water was available for the following 24 h. As we have previously not observed PGC‐1α‐dependent gender differences in metabolic parameters (Leick et al. 2008), female mice were used in the present study due to availability. Fed and fasted mice were euthanized by cervical dislocation at the end of the 24 h intervention period and quadriceps muscles were rapidly excised and snap frozen in liquid nitrogen. Quadriceps muscle was chosen to ensure sufficient amount of tissue for the analyses. Trunk blood was obtained in EDTA containing tubes and plasma was obtained after centrifugation. Both muscle and plasma were stored at −80°C. A subset of mice (*n* = 8–10; individually housed) were acclimatized to the environment of the cabinets in a TSE Phenomaster unit (TSE Systems, Bad Hamburg, Germany) for 3 days with a constant setting of 22°C, 30% humidity, and a 12:12‐h light–dark cycle for determination of activity level and indirect calorimetry measurements followed by data collection (laser beam breaks, respiratory exchange ratio [RER]) for 24 h with ad libitum access to food and water followed by 24 h of fasting with only access to water.

### Plasma analyses

Plasma free fatty acid concentrations were measured using a NEFA‐HR kit according to the manufacturer's guidelines (WAKO Diagnostics GmbH, Germany). Plasma glucose and lactate were measured fluorometrically as described previously (Lowry and Passonneau 1972).

### Muscle analyses

Whole quadriceps muscles were crushed in liquid nitrogen to achieve tissue homogeneity. For measurements of muscle glucose, lactate, and glucose‐6‐phosphate (G‐6‐P), 10–15 mg of crushed muscle tissue was extracted in perchloric acid (PCA), neutralized to a pH of 7–8 and measured as described above for plasma. Muscle glycogen was determined fluorometrically as glycosyl units after hydrolyzing 10–15 mg wet weight muscle samples by boiling for 2 h in 1 mol/L HCl as described previously (Lowry and Passonneau 1972).

### Immunoblotting

Crushed muscle samples (25–30 mg) were homogenized in lysis buffer (10% glycerol, 20 mmol/L Na‐pyrophosphate, 150 mmol/L NaCl, 50 mmol/L HEPES, 1% NP‐40, 20 mmol/L *β*‐glycerophosphate, 10 mmol/L NaF, 1 mmol/L EDTA, 1 mmol/L EGTA, 20 *μ*g/ml aprotinin, 10 *μ*g/ml leupeptin, 2 mmol/L Na_3_VO_4_, 3 mmol/L benzamidine, and deacetylase inhibitors [nicotinamide, 1 mmol/L, and sodium butyrate, 5 mmol/L], pH 7.5) using a Tissue Lyser II (Qiagen, Germany). Protein concentration in each of the samples was determined using the bicinchoninic acid method (Thermo Fischer Scientific) and protein concentration was adjusted with sample buffer to a concentration of 1 *μ*g/*μ*l. Protein phosphorylation and protein content were determined by SDS‐PAGE using hand‐casted gels and western blotting. PVDF membranes were incubated in optimized primary antibody solutions overnight at 4°C for determination of AMPK*α*2 (1:20,000), PDK4 (1:4000), and PDH‐E1*α* protein (1:1000), PDH‐E1*α* ^Ser293^(1:1000), PDH‐E1*α* ^Ser300^ (1:1000), and PDH‐E1*α* ^Ser295^phosphorylation (1:1000) (all kindly provided by Professor Grahame Hardie, University of Dundee, Scotland), hexokinase (HK) II protein (1:1000), AMPK^Thr172^phosphorylation (1:1000), total lysine acetylation (1:1000) (#2867, #2535 and #9441, respectively, Cell Signaling Technologies, Danvers, MA), acetyl‐CoA carboxylase (ACC)^Ser212^ phosphorylation (1:1000), and PDH‐E1*α* ^Ser232^ phosphorylation (1:4000) (07‐303 and #AP1063, respectively, EMD Millipore, Bedford), TBC1D4 (1:20.000), PDK1 (1:1000), and OXPHOS proteins (1:1000) (ab24469, ab90444, and ab110413, respectively, Abcam, Cambridge, UK), PDK2 protein (1:1000) (ST1643, CalBioChem, Bedford), PDP1 protein (1:1000) (Sigma‐Aldrich, St. Louis), and GLUT4 protein (1:1000) (PAI‐1065, ABR, Connecticut). Species‐specific horseradish peroxidase‐conjugated immunoglobulin secondary antibodies (DAKO, Denmark) were used for incubation the following day. ACC2 protein was detected using streptavidin (1:2000) (Dako, Glostrup, Denmark). Protein bands were subsequently visualized using an ImageQuant LAS 4000 imaging system and quantified with ImageQuant TL 8.1 software (GE Healthcare, Freiburg, Germany).

### Immunoprecipitation and PDH‐E1*α* acetylation

A total of 200 *μ*g protein from lysate was immunoprecipitated for the determination of global PDH‐E1*α* acetylation. Briefly, the lysate was added to PBS‐rinsed protein G agarose beads (EMD Millipore, Bedford) in a 50:50 solution with PBS buffer containing 0.5% Triton X with 2 *μ*g of PDH‐E1*α* antibody. The samples were rotated end over end at 4°C overnight and on the subsequent day the beads were washed, sample buffer was added, and the samples were heated at 96°C for 3 min. The beads were spun down to avoid transfer and lysate loaded on a hand‐casted gel for SDS‐page and western blotting as described above. For each sample, acetylated protein was normalized to the amount of precipitated PDH‐E1*α* protein content determined by western blotting.

### PDHa activity

PDHa activity was determined after homogenizing 10–15 mg of wet weight muscle tissue and snap‐freezing the homogenate in liquid nitrogen as described previously (Cederblad et al. 1990; Constantin‐Teodosiu et al. 1991; Putman et al. 1993) with modifications (Pilegaard et al. 2006) and PDHa activity was normalized to creatine content in each muscle sample to correct for nonmuscle tissue in the sample as previously described (St Amand et al. 2000).

### Statistics

All values are expressed as means ± standard error. A two‐way ANOVA was applied to test the effects of genotype and intervention. If data passed the prerequisites of normality and equal variance and a main effect was detected, a Student–Newman–Keuls test was applied as a post hoc test to locate specific differences in group means (McHugh 2011). For single grouped data, a Student's *t*‐test was used to test if a difference was present. Significance was accepted at *P*s < 0.05. The statistical tests were performed using Sigmaplot 13.0 (Systat, San Jose, CA, USA).

## Results

### Metabolic markers

**Figure 1:**
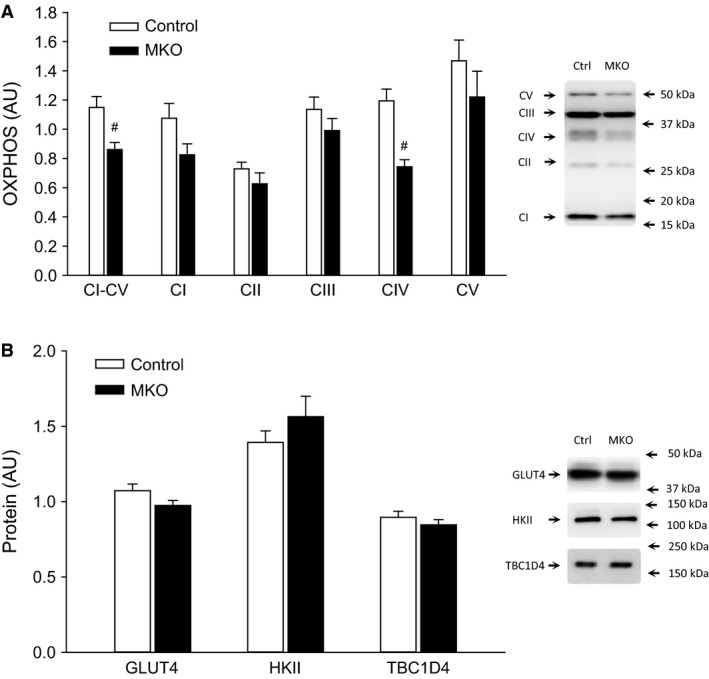
(A) Skeletal muscle total OXPHOS combined complex average and individual complexes I–V and (B) GLUT4, HKII, and TBC1D4 protein content from fed (FED) and 24 h fasted (FAST) skeletal muscle‐specific PGC‐1*α* knockout (MKO) and littermate‐floxed control (control) mice. Values are given as mean ± SE (*n* = 11). Protein levels are given in arbitrary units (AU). *Significantly different from FED within given genotype (*P* < 0.05). #Significantly different from control within given group (*P* < 0.05).

OXPHOS basal protein content was lower (*P* < 0.05) in PGC‐1*α* MKO than control mice. There were no significant differences in basal HKII, GLUT4, and TBC1D4 protein between genotypes (Fig. 1).

### Indirect calorimetry and locomotor activity

**Figure 2:**
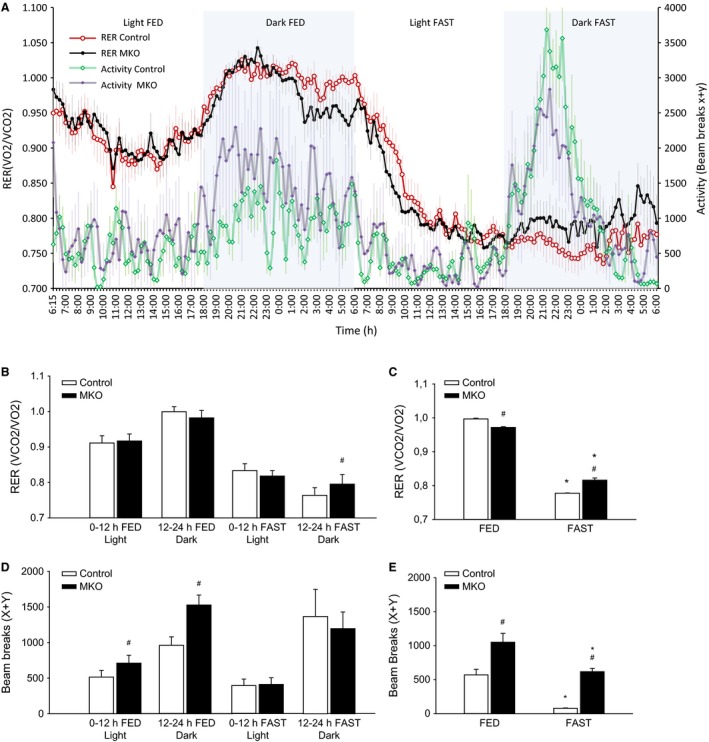
(A) Forty‐eight hours of continuous respiratory exchange ratio (RER) and locomotor activity. (B) Average RER divided into light and dark phases of both the 24 h fed and 24 h fasting intervention. (C) Average RER in the last hour of the fed and the fasted state. (D) Average locomotor activity divided into light and dark phases of both the 24 h fed and 24 h fasting intervention. (E) Average locomotor activity in the last hour of the fed and the fasted state in skeletal muscle‐specific PGC‐1*α* knockout (MKO) and littermate‐floxed control (control) mice. Values are given as mean ± SE (*n* = 8–10). *Significantly different from FED within given genotype (*P* < 0.05). #Significantly different from control within given group (*P* < 0.05).

RER was overall lower (*P* < 0.05) and activity higher (*P* < 0.05) in PGC‐1*α* MKO than control mice at night in the fed state, while RER was overall higher (*P* < 0.05) and activity lower (*P* < 0.05) in PGC‐1*α* MKO than control mice at night in the fasted state (Fig. 2A and B). In the final hour of the dark, fed period (5–6 am), RER was lower (*P* < 0.05) and in the last hour of the dark, fasting period higher (*P* < 0.05) in PGC‐1*α* MKO than control mice (Fig. 2C).

### Plasma glucose and NEFA

The plasma free fatty acid concentration increased (*P* < 0.05) and the plasma glucose concentration decreased (*P* < 0.05) in both PGC‐1*α* MKO and control mice with fasting. There were no differences in either plasma NEFA or plasma glucose levels between genotypes (Table 1).

**Table 1:** Plasma glucose and nonesterified fatty acids (NEFA) in fed (FED) and 24 h fasted (FAST) skeletal muscle‐specific PGC‐1*α* knockout (MKO) and littermate‐floxed control (control) mice

	FED		FAST	
	Control	MKO	Control	MKO
Glucose (mmol/L)	6.11 ± 0.2	6.03 ± 0.2	4.03 ± 0.17*	4.24 ± 0.22*
NEFA (mmol/L)	0.15 ± 0.01	0.13 ± 0.01	0.23 ± 0.01*	0.25 ± 0.02*

Values are given as mean ± SE (*n* = 8–10).

*Significantly different from FED within given genotype (*P* < 0.05).

### Muscle metabolites

Muscle glucose and glycogen concentrations decreased (*P* < 0.05) in both PGC‐1*α* MKO and control mice in response to fasting with no difference between genotypes. G‐6‐P decreased (*P* < 0.05) with fasting in PGC‐1*α* MKO and tended to be lower (0.1 ≤ *P* ≤ 0.05) in PGC‐1*α* MKO mice than control mice (Table 2).

**Table 2:** Concentrations of skeletal muscle glucose, glucose‐6‐phosphate (G‐6‐P), and glycogen in fed (FED) and 24 h fasted (FAST) skeletal muscle‐specific PGC‐1*α* knockout (MKO) and littermate‐floxed control (control) mice

	FED		FAST	
	Control	MKO	Control	MKO
Glucose (mmol/kg)	0.84 ± 0.06	0.77 ± 0.06	0.47 ± 0.04*	0.48 ± 0.03*
G‐6‐P (mmol/kg)	2.37 ± 0.17	2.20 ± 0.11	1.97 ± 0.35	1.40 ± 0.18*
Glycogen (mmol/kg)	23.9 ± 2.3	24.5 ± 1.6	15.9 ± 1.6*	13.0 ± 1.5*

Values are given as mean ± SE (*n* = 11).

*Significantly different from FED within given genotype (*P* < 0.05).

^#^Significantly different from control within given group (*P* < 0.05).

### AMPK and ACC

**Figure 3:**
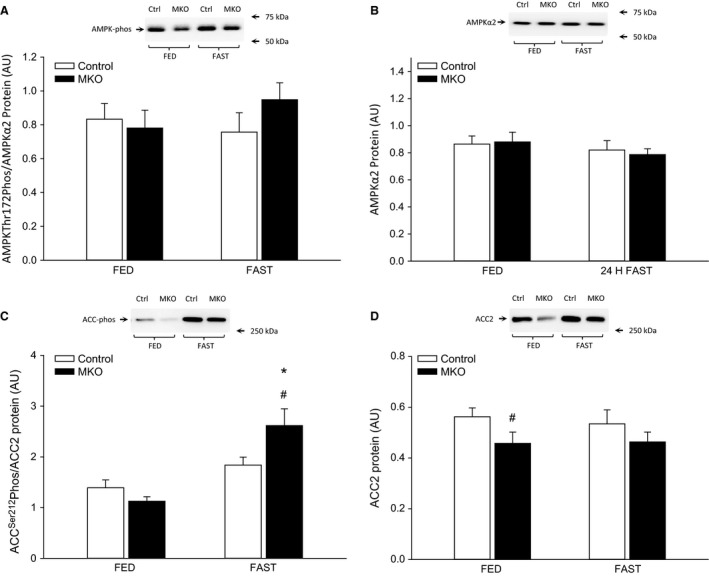
(A) AMPK Thr172 phosphorylation normalized to AMPK *α*2 protein content, (B) AMPK *α*2 protein content, (C) acetyl‐CoA carboxylase 2 (ACC2) Ser212 phosphorylation normalized to ACC2 protein content, (D) ACC2 protein content in quadriceps from fed (FED) and 24 h fasted (FAST) skeletal muscle‐specific PGC‐1*α* knockout (MKO) and littermate floxed controls (control) mice. Values are given as mean ± SE (*n* = 11). Protein and phosphorylation (phos) levels are given in arbitrary units (AU). *Significantly different from FED within given genotype (*P* < 0.05). #Significantly different from control within given group (*P* < 0.05).

There were no differences in either absolute (Fig. 3A) or normalized AMPK phosphorylation or in AMPK*α*2 protein in skeletal muscle with fasting or between genotypes (Fig. 3B). There was a tendency for an increase (0.05 ≤ *P* ≤ 0.1) in absolute ACC phosphorylation (Fig. 3C) with fasting in control mice, while there was no difference in normalized ACC phosphorylation with fasting in control mice (Fig. 3D). Fasting increased (*P* < 0.05) both absolute (Fig. 3C) and normalized ACC phosphorylation (Fig. 3D) in skeletal muscle of PGC‐1*α* MKO mice, while ACC2 protein was lower (*P* < 0.05) in PGC‐1*α* MKO than control mice in the fed state.

### PDHa activity and PDH‐E1*α* protein

**Figure 4:**
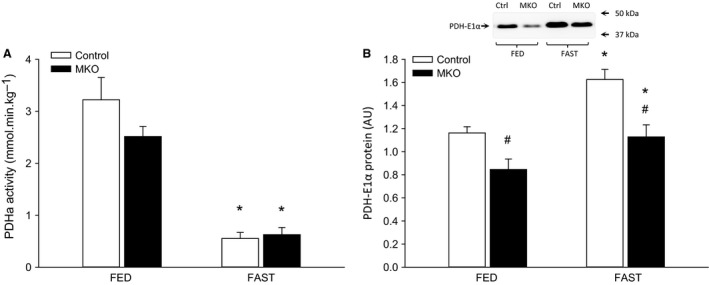
(A) PDHa activity and (B) PDH‐E1*α* protein in quadriceps muscle from fed (FED) and 24 h fasted (FAST) skeletal muscle‐specific PGC‐1*α* knockout (MKO) and littermate‐floxed controls (control) mice. Values are given as mean ± SE (*n* = 11). Protein levels are given in arbitrary units (AU). *Significantly different from FED within given genotype (*P* < 0.05). #Significantly different from control within given group (*P* < 0.05).

PDHa activity decreased (*P* < 0.05) markedly in skeletal muscle with 24 h of fasting in both PGC‐1*α* MKO and control mice with no difference between genotypes (Fig. 4A). PDH‐E1*α* protein content increased (*P* < 0.05) in both genotypes after 24 h of fasting relative to fed and PDH‐E1*α* protein was lower (*P* < 0.05) in PGC‐1*α* MKO than control mice in both the fed and fasted state (Fig. 4B).

### PDH phosphorylation

**Figure 5:**
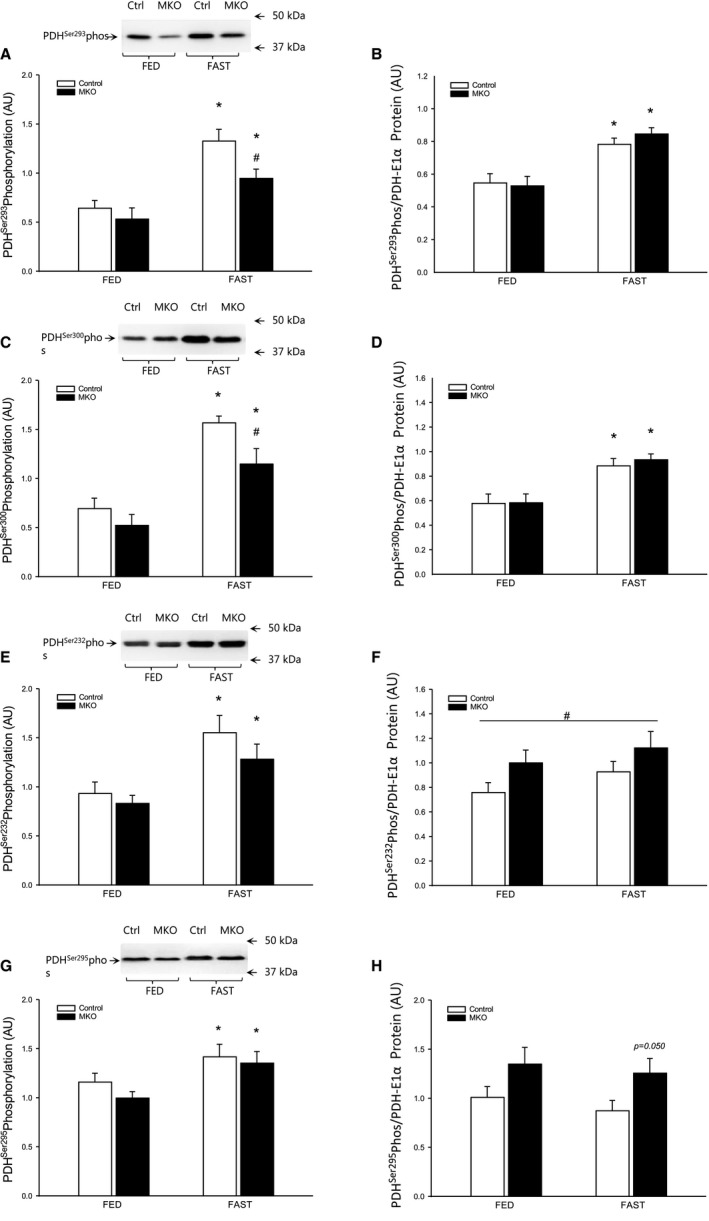
(A) PDH Ser293 phosphorylation, (B) PDH Ser293 phosphorylation normalized to PDH‐E1*α* protein content, (C) PDH Ser300 phosphorylation, (D) PDH Ser300 phosphorylation normalized to PDH‐E1*α* protein content, (E) PDH Ser232 phosphorylation, (F) PDH Ser232 phosphorylation normalized to PDH‐E1*α* protein content, (G) PDH Ser295 phosphorylation, (H) PDH Ser295 phosphorylation normalized to PDH‐E1*α* protein content in quadriceps muscle from fed (FED) and 24 h fasted (FAST) skeletal muscle‐specific PGC‐1*α* knockout (MKO) and littermate‐floxed controls (control) mice. Values are given as mean ± SE (*n* = 11). Protein and phosphorylation (phos) levels are given in arbitrary units (AU). *Significantly different from FED within given genotype (*P* < 0.05). #Significantly different from control within given group (*P* < 0.05).

PDH^Ser293^, PDH^Ser300^, PDH^Ser232^, and PDH^Ser295^ phosphorylation increased (*P* < 0.05) with 24 h of fasting, regardless of genotype. PDH^Ser293^ and PDH^Ser300^ phosphorylation was lower (*P* < 0.05) in PGC‐1*α* MKO than control mice in the fasted state (Fig. 5A, C, E, G). When normalized to PDH‐E1*α* protein, phosphorylation sites PDH^Ser293^ and PDH^Ser300^ increased (*P* < 0.05) to a similar level with fasting in PGC‐1*α* MKO and control mice, while PDH^Ser232^ overall was higher (*P* < 0.05) in PGC‐1*α* MKO than control mice and PDH^Ser295^ tended to be higher (0.05 ≤ *P* ≤ 0.1) in PGC‐1*α* MKO than control mice both in the fed state and after 24 h of fasting (Fig. 5B, D, F, H).

### PDK1, 2, 4, and PDP1

**Figure 6:**
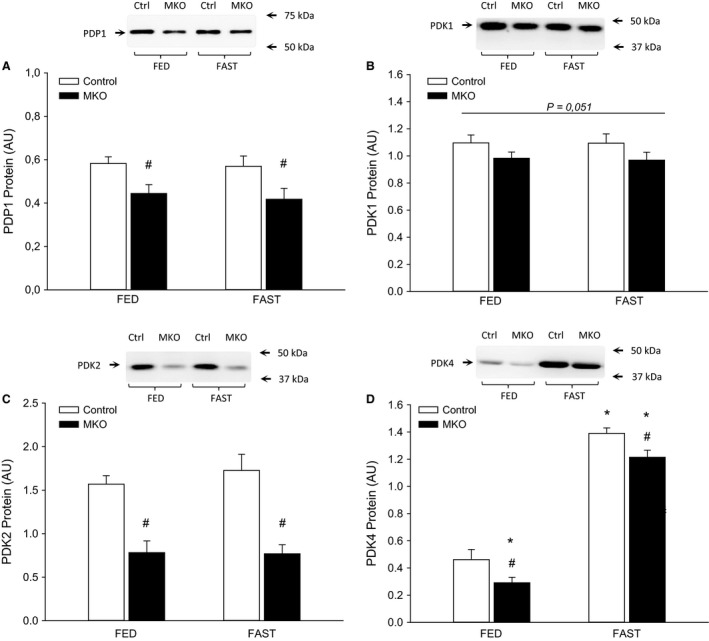
(A) PDP1 protein content, (B) PDK1 protein content, (C) PDK2 protein content, (D) PDK4 protein content in quadriceps muscle from fed (FED) and 24 h fasted (FAST) skeletal muscle‐specific PGC‐1*α* knockout (MKO) and littermate floxed controls (control) mice. Values are given as mean ± SE (*n* = 11). Protein levels are given in arbitrary units (AU). *Significantly different from FED within given genotype (*P* < 0.05). #Significantly different from control within given group (*P* < 0.05).

There was an overall tendency for lower (*P* < 0.05) skeletal muscle PDK1 protein in PGC‐1*α* MKO than control mice, while PDP1 protein and PDK2 protein were lower (*P* < 0.05) in PGC‐1*α* MKO than control mice with no effect of the fasting intervention (Fig. 6A–C). PDK4 protein increased (*P* < 0.05) in both genotypes after 24 h of fasting and was lower (*P* < 0.05) in PGC‐1*α* MKO than control mice both in the fed and the fasted state (Fig. 6D).

### SIRT3 and PDH acetylation

**Figure 7:**
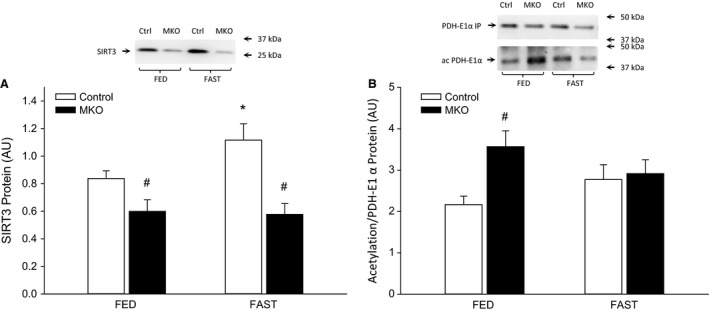
(A) SIRT3 protein content and (B) total lysine acetylation of immunoprecipitated PDH‐E1*α* protein in quadriceps muscle from fed (FED) and 24 h fasted (FAST) skeletal muscle‐specific PGC‐1*α* knockout (MKO) and littermate floxed controls (control) mice. Values are given as mean ± SE (*n* = 11). Protein and acetylation levels are given in arbitrary units (AU). *Significantly different from FED within given genotype (*P* < 0.05). #Significantly different from control within given group (*P* < 0.05).

Skeletal muscle SIRT3 protein was lower (*P* < 0.05) in PGC‐1*α* MKO than control mice in the fed state and increased (*P* < 0.05) after 24 h of fasting only in control mice (Fig. 7A). PDH‐E1*α* acetylation was higher (*P* < 0.05) in PGC‐1*α* MKO than control mice in the fed state, but was not different between genotypes after 24 h of fasting (Fig. 7B).

## Discussion

The main findings of the present study are that lack of muscle PGC‐1*α* did not affect the switch from CHO to fat utilization in the transition from the fed to the fasted state, but was associated with higher CHO use in the fasted state potentially influencing the ability to endure prolonged starvation. Fasting‐induced downregulation of PDHa activity in skeletal muscle of control mice was associated with increased phosphorylation of all four known sites in PDH‐E1*α* as well as with increased PDK4 and SIRT3 protein without changes in total acetylation of PDH‐E1*α*. Lack of muscle PGC‐1*α* reduced PDH‐E1*α*, PDK1, 2, 4, PDP1, and SIRT3 protein content as well as increased total lysine PDH‐E1*α* acetylation in the fed state. Knockout of muscle PGC‐1*α* did not influence the fasting‐induced increase in PDH‐E1*α* phosphorylation, but prevented the fasting‐induced increase in SIRT3 protein, while additional factors seem to override PGC‐1*α‐*mediated regulation of PDH‐E1*α* acetylation during fasting.

The observed switch in substrate utilization from CHO to fat and the lower RER value during fasting than in the fed state in the control mice are in accordance with numerous previous studies (Kersten et al. 1999; Frier et al. 2011; Jensen et al. 2013; Wueest et al. 2014) demonstrating an experimental foundation for examining the impact of skeletal muscle PGC‐1*α* on the ability to switch substrate during fasting. The present finding that lack of skeletal muscle PGC‐1*α* had no clear impact on the time course of the change in RER during the switch from the fed to the fasted state suggests that skeletal muscle PGC‐1*α* is not required for metabolic flexibility during this transition. This is as such in accordance with the previous observation that lack of muscle PGC‐1*α* did not influence the fluctuations in RER over the course of 24 h with access to food (Finley et al. 2012).

Because the activity level was higher in PGC‐1*α* MKO than control mice during the dark period in the fed state, and increased activity is expected to enhance carbohydrate utilization, the genotype difference in substrate oxidation in the fed state does not seem to be related to activity differences. Likewise, the differences in average activity levels between genotypes in the fed state, and generally high levels of activity in the fasted state, were not associated with differences in RER values. That being said, differences in activity level cannot be completely excluded to have an effect on the observed RER differences. Moreover, the similar plasma glucose and NEFA concentrations as well as similar muscle glycogen in fed PGC‐1*α* MKO and control mice indicate that the RER differences in the fed state are not due to differences in substrate availability between the genotypes.

The observation that PGC‐1*α* MKO mice had higher RER in the final phase of the fasted state than the control mice indicates that lack of skeletal muscle PGC‐1*α* reduced fat oxidation in the fasted state, although this effect cannot be excluded to be driven by differences in locomotor activity. The finding that the PGC‐1*α* MKO mice overall were less physically active than the controls during the last 12 h of fasting shows that the higher carbohydrate oxidation was not due to increased activity level in the PGC‐1*α* MKO mice relative to control. Furthermore, the similar plasma glucose and NEFA concentrations as well as muscle glycogen level in the two genotypes suggests that differences in substrate availability did not elicit the difference in substrate utilization. However, while the decrease in plasma glucose and the increase in plasma NEFA concentration were independent of genotype, the finding that the absolute decrease in mean muscle glycogen was 44% greater in PGC‐1*α* MKO than control mice supports the higher reliance on carbohydrates in the PGC‐1*α* MKO than the control mice. Based on the previous observation that muscle‐specific PGC‐1*α* overexpression resulted in reduced glycogen phosphorylase content and phosphorylation (Wende et al. 2007) with concomitantly reduced glycogen use during exercise, it may be suggested that the higher glycogen use in PGC‐1*α* MKO mice in the present study is caused by an elevated glycogen phosphorylase activity. Furthermore, the observed decrease in skeletal muscle G‐6‐P with fasting in PGC‐1*α* MKO mice only supports that the glycolytic flux and glucose oxidation were higher in PGC‐1*α* MKO than control mice. In addition, the reduced fat oxidation in PGC‐1*α* MKO mice relative to control mice in the fasted state may imply that low skeletal muscle PGC‐1*α* content is associated with impaired carbohydrate sparing during food deprivation. The present observation that TBC1D4 protein content was similar in the two genotypes may suggest that the higher carbohydrate oxidation in PGC‐1*α* MKO mice was not due to differences in GLUT4 translocation to the plasma membrane, although it cannot be ruled out that other factors in the translocation process may be affected by lack of PGC‐1*α*.

The present finding that SIRT3 protein increased with 24 h of fasting in control mice is in agreement with previous findings (Palacios et al. 2009; Hirschey et al. 2010; Caton et al. 2011). Previous studies suggest that ROS balance is impaired when PGC‐1*α* is lacking (St‐Pierre et al. 2006; Adhihetty et al. 2009) and that lack of PGC‐1a furthermore leads to ROS accumulation (St‐Pierre et al. 2006; Olesen et al. 2013). ROS has been shown to increase in mouse skeletal muscle with fasting (Qi et al. 2014; Rahman et al. 2014) and as SIRT3 has been shown to play a role in ROS scavenging (Qiu et al. 2010; Yu et al. 2012) increasing SIRT3 protein may be an adaptation to handling a prolonged period of increased ROS production. SIRT3 protein has previously been shown to increase with fasting and caloric restriction in wild‐type mice (Palacios et al. 2009) and to increase with both repeated AICAR injections and exercise training in a PGC‐1*α*‐dependent manner using PGC‐1*α* KO mice (Brandauer et al. 2015). The present observation that skeletal muscle SIRT3 protein was lower in PGC‐1*α* MKO mice than controls is therefore in accordance with previous studies. However, the present finding that SIRT3 protein content was completely unresponsive to 24 h of fasting in PGC‐1*α* MKO mice is novel. Furthermore, the higher lysine acetylation level of PDH‐E1*α* in PGC‐1*α* MKO mice than controls in the fed state is in agreement with a previous study using myocytes (Jing et al. 2013) and with the present genotype difference in SIRT3 protein content. The lack of a genotype difference in PDH‐E1*α* acetylation in the fasted state indicates on the other hand that PGC‐1*α*‐deficient mouse skeletal muscle is capable of maintaining the PDH acetylation state equal to control muscle despite reduced SIRT3 protein level. As muscle NAD+ levels have been shown to increase with fasting (Canto et al. 2010), this may upregulate SIRT3 activity (Schwer et al. 2002) sufficiently in PGC‐1*α* MKO mice to obtain an acetylation state as in control mice.

The present observation that 24 h of fasting led to robust increases in absolute phosphorylation of all the four PDH phosphorylation sites, PDH^Ser293^, PDH^Ser300^, PDH^Ser232^, and PDH^Ser295^, in both genotypes is in line with the downregulation of PDHa activity and the previously reported fasting effects in PGC‐1a KO and PGC‐1a MCK mice (Kiilerich et al. 2010). On the other hand, the fasting‐induced increase in skeletal muscle PDH^Ser232^ phosphorylation has not been reported previously. The observed increase in PDH‐E1*α* protein with fasting was unexpected, but might be explained by a possible increase in PGC‐1*α* and thus mitochondrial content as previously reported in liver (Haase et al. 2011) and skeletal muscle (Palacios et al. 2009; Canto et al. 2010). However, the lack of a similar increase in OXPHOS protein with fasting in the present study (data not shown) does not support this notion. Regardless, it appears that PGC‐1*α* is partly dispensable for fasting‐induced increases in PDH‐E1*α* protein.

PDH phosphorylation levels were also normalized to PDH‐E1*α* protein content due to the impact of both genotype and intervention on PDH‐E1*α* protein content. The observation that normalization of PDH phosphorylation to PDH‐E1*α* protein resulted in similar or even higher levels of PDH phosphorylation in PGC‐1*α* MKO than control mice indicates a maintained capability to regulate PDH relative to the amount of PDH‐E1*α* protein despite lack of PGC‐1*α*. Furthermore, the small genotype differences in both site PDH^Ser232^ and PDH^Ser295^ when normalized to PDH‐E1*α* protein may be due to the site‐specific affinity of the different PDK isoforms as previously reported (Korotchkina and Patel 2001; Patel and Korotchkina 2001).

The finding that skeletal muscle PDK4 protein was markedly increased with fasting is in agreement with numerous previous studies in humans, rats, and mice (Wu et al. 1999, 2001; Pilegaard et al. 2003; Spriet et al. 2004; Zhang et al. 2006; Kiilerich et al. 2010). Although PDK4 protein levels were lower in PGC‐1α MKO than control mice both in the fed and the fasted state, the same absolute increase in PDK4 protein was evident in both genotypes underlining that skeletal muscle PGC‐1α plays a role in basal PDK4 expression, but is not necessary for the observed fasting‐induced upregulation of PDK4 protein content. The finding that the protein content of PDP1, PDK1, and PDK2 were unaffected by fasting is in accordance with previous studies (Spriet et al. 2004; Bienso et al. 2014), but the lower protein levels of PDP1, PDK1, and PDK2 in PGC‐1*α* MKO mice than controls have not been reported previously. This observation, in conjunction with reduced skeletal muscle OXPHOS and PDH‐E1*α* content in PGC‐1a MKO mice, as also previously shown in PGC‐1*α* KO mice (Kiilerich et al. 2010), indicates a reduced capacity for regulation of glucose oxidation. However, the present observation that the PDHa activity did not differ between genotypes suggests that additional factors influence PDHa activity. In addition, the observation that the genotype differences in RER were not reflected in differences in skeletal muscle in vitro PDHa activity supports the previous findings that there can be a dissociation between PDHa activity and actual flux through the PDC (Constantin‐Teodosiu et al. 2004).

Skeletal muscle AMPK phosphorylation has been reported to increase in rodents with shorter fasting protocols (De et al. 2006; Canto et al. 2010; Frier et al. 2011). However, the present finding of no change in AMPK phosphorylation indicates that AMPK does not exhibit increased phosphorylation levels after 24 h of fasting as reported previously in mice (Gonzalez et al. 2004) and humans (Wijngaarden et al. 2014). It cannot be excluded that the mice in the present study had transiently increased AMPK activation during the fasting intervention in the hours prior to euthanization, and that this activation differed between genotypes, especially with differing locomotor activity in the light phase of both the fed and fasting intervention. The observed increase in phosphorylation of ACC with fasting likely contributing to the enhanced fatty acid oxidation (FAO) might reflect a preceding, transient activation of AMPK as previously observed in rat skeletal muscle after 12 h of fasting, but not 48 h of fasting (De et al. 2006). On the other hand, ACC deactivation may also occur through a proposed fasting‐induced *β*‐adrenergic epinephrine/PKA axis (Frier et al. 2011). The finding that ACC phosphorylation was higher in PGC‐1*α* MKO mice than controls during fasting when normalized to protein content may indicate fewer active ACC proteins and hence less malonyl CoA to inhibit lipid uptake into the mitochondria in PGC‐1*α* MKO than control mice. Furthermore, the observed similar FFA plasma level in the two genotypes suggests a similar delivery of fatty acids to the muscles in the two genotypes. These observations are therefore not in accordance with the higher RER values indicating elevated CHO oxidation in PGC‐1*α* MKO mice than controls. However, both CD36 mRNA and CPT1 mRNA content have been shown to increase with fasting (Long et al. 2005; Frier et al. 2011) and to be PGC‐1*α* dependent (Calvo et al. 2008) suggesting that the mitochondrial fatty acid transport step may limit fat oxidation during fasting in PGC‐1*α* deficient muscle and hence potentially explain the reduced fat oxidation during fasting when PGC‐1*α* is lacking.

In summary, the present study suggests that skeletal muscle PGC‐1*α* plays an important role in skeletal muscle mitochondrial protein adaptations to prolonged fasting, as seen with PDH‐E1*α* and SIRT3, but is dispensable for maintaining metabolic flexibility in the transition from the fed to the fasted state. Furthermore, lack of skeletal muscle PGC‐1*α* reduces the content of PDH regulatory proteins and PDH‐E1*α* protein as well as alters PDH phosphorylation and the acetylation pattern in skeletal muscle. In addition, while both fed and fasting‐induced PDHa activities appear independent of PGC‐1α, PGC‐1α is required for fasting‐induced SIRT3 protein upregulation in skeletal muscle.

## Conflict of Interest

None declared.
